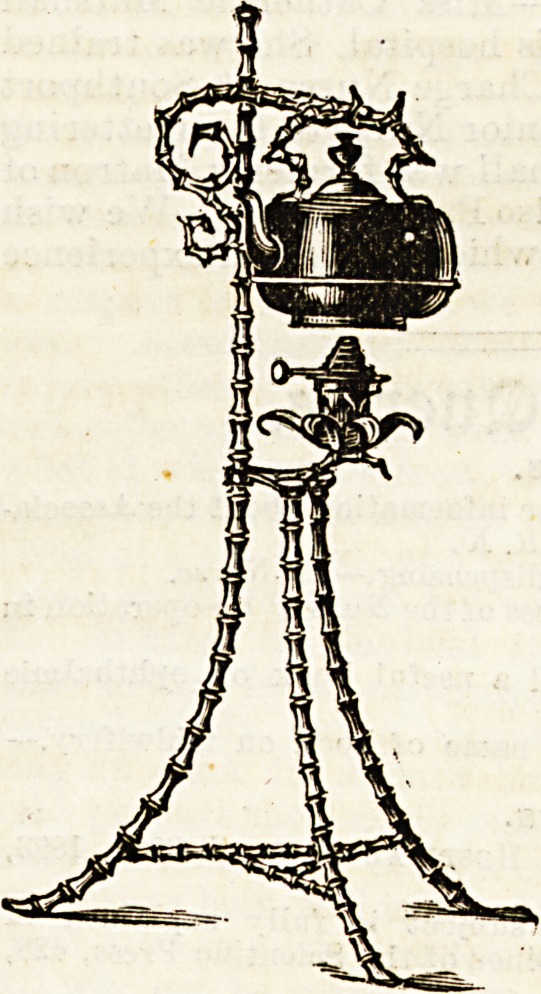# Extra Supplement.—The Nursing Mirror

**Published:** 1893-11-04

**Authors:** 


### "The Hospital" Nursing Mirror.

Being the Extra Nursing Supplement of "The Hospital" Newspaper.

[Contributions for this Supplement should be addressed to the Editor, The Hospital, 428, Strand, London, W.C., and should have the word "Nursing" plainly written in left-hand top corner of the envelope.]

### News from the Nursing World.

### "WAIT FOR AN ANSWER."

"I want to know" is a familiar introductory sentence to requests for information about nursing in every quarter of the globe which reach The Hospital office. That each letter ends with polite apologies for "giving trouble" goes without saying. It is equally certain that the answering of such questions is not done grudgingly; on the contrary, the letters from Japan in to-day's issue, and previous ones from different parts of Africa, the U.S.A., California, India, Newfoundland, and many other places, show that there is a laudable desire on the part of nurses to give fellow-workers the benefit of their experience. We wish our readers clearly to understand that questions are always welcome, and if they give time enough for answers to be received, even from distant colonies or foreign stations, in all of which The Hospital circulates, we can secure for them such information as they may desire.

### ASK THE DOCTOR.

"First the doctor, then the nurse" is the appropriate sentiment embodied in two excellent letters which *Nursing Notes* publishes in this month's number, written by two Queen's nurses. They give throughout most complete evidence of sound "training" and also of much common sense. The original epistle which provoked these replies appeared in the October issue of *Nursing Notes*, signed by one of the North London Association District nurses, who gives this extraordinary counsel to her readers: "Do not seek advice from a doctor on the subject of an ulcer," which, she further states, "is not a wound"! Small wonder that a F.R.C.S. has written a clever and sarcastic little homily on this somewhat unique text. Such utter ignorance of the subordinate position which a nurse holds towards doctors is still further evidence of the necessity for only fully-trained women being placed in the responsible position of district nurses. From them, at any rate, we have a right to expect sounder doctrines.

### NOT ALL BY RULE.

"I suppose you have to keep to rules, Nurse?" asked a new patient. "Certainly, I have, but there is a reason for most of them," responded the nurse. The patient lay for a while reflecting in silence that her fears would soon be realised. She had always been warned that trained nurses had "hard and fast" rules about everything, and now she must personally experience the discipline, and she sighed at the prospect. Presently the nurse spoke again, standing at the bedside with a basin of warm water ready to make her patient's toilet for the night. "What temperature is the water that I am to be washed in?" said the invalid nervously. "It shall be just whatever is agreeable to you," answered the surprised nurse. "Then you haven't rules for everything after all?" and the patient's tone of relief nearly upset the nurse's gravity. "When is my poultice to be changed?" was the next inquiry. "Very soon." "Shall you put it on very hot?" said the patient, with a fresh accession of "nerves." "The temperature shall be what you like." "Oh, dear me, that is really a comfort! I thought after you had taken the poultice's temperature with your thermometer I should have to bear it without a word." At this moment nurse found it convenient to turn her face away from the invalid, and directed an uncontrollable smile on the nearest fireplace.

### IS THERE ANY CHARGE?

This is one of the questions which is apt to accompany letters to the Editor enclosing notices of appointments, &c. All such insertions are free, and are found to give much pleasure to our readers, who often thus hear of old fellow-workers, from whom they have, perhaps, been parted for years. The only conditions attending the publication of appointments being that they should always be accompanied by copies of testimonials, and reports of previous posts held, as well as particulars of training.

### WOMEN WORKERS.

On October 27th a meeting of the members of the Central Conference Council of the National Union of Women Workers took place at the Windsor Hotel, Victoria Street. For this lengthy title "the three C's" was conveniently substituted by Miss Hubbard in her introductory speech. Many subjects were brought forward, and the meeting was well attended and interesting. The difficulties of securing proper midday meals by ladies working in offices was discussed at some length, a few speakers considering the prices of refreshments at respectable restaurants prohibitive, whilst others mentioned various places where moderate charges were the rule. Persons who dogmatise on the supposed evils of tea drinking would have rejoiced to hear how many women now add a glass of plain hot water to their meals; in fact, this somewhat unattractive beverage has become so popular that there is a fixed charge made for it at places where it used to be provided gratis. The students at the National Gallery seem somewhat universally troubled by symptoms of indigestion, and numbers of them favour this harmlessly insipid beverage. Active nurses are happily but little troubled by those ailments which render miserable the lives of persons engaged in sedentary pursuits. Amongst the announcements made at this C.C.C. meeting was one received with great applause, to the effect that Miss Florence Nightingale had promised a paper on Health Teaching in Villages, to be read by Mrs. Verney, at the Conference at Leeds on November 7th.

### NURSES' HOME AND CLUB.

The Duchess of Portland has promised to open the new Nurses' Club at 17, Nottingham Place, in the first week in December. The house is conveniently situated and exceedingly comfortable, and nurses can be accommodated by the day or the week at fixed charges. They can have separate rooms or cubicles, according to their tastes and means. There is a large general sitting-room for residents, and a recreation-room which will be open to all the club members. Here for an annual subscription they will be free to meet friends, have tea, &c. Private nurses who do not live in the house can arrange to have their letters received and forwarded by paying an annual subscription. The recreation-room has been furnished by friends, and will be maintained by the nurses own subscriptions. All particulars can be obtained at the Home, or of Mrs. Stubbs, Beresford House, Beaumont Street, W.

### AT LAST.

At last there seems some chance of the infirm poor receiving skilled care. We have long felt that the inmates of sick wards in workhouses, which are quite distinct from poor-law infirmaries, were being overlooked in the midst of other nursing reforms. However, a practical step forward has been taken by Lady Meath. At a meeting held to consider this subject on October 23rd, at her own house, she offered to pay for the first six trained nurses supplied to the helpless and infirm inhabitants of workhouses. This is progress in the right direction, but as yet the supply of qualified nurses has proved most inadequate to meet the demands of even the workhouse infirmaries, where there is more variety of work. Apparently there is a strong prejudice against infirmaries in the mind of many trained nurses. Directly the subject is brought forward objections are invariably raised very quickly. These protests being often made by earnest hard workers, there must be something in them. Perhaps it is the large number of patients for which each nurse is responsible, or the fact that when she goes off duty she takes her anxieties with her, that act deterrently. The responsibilities of the charge nurse in a hospital end when she goes out of her ward, another equally competent worker taking her place. But very often the workhouse infirmary nurse has nothing but the rawest of assistants to leave in charge, and therefore she has to say, "Come to my room if anything is wanted," when she goes away for a short interval, and therefore is often nominally only "off duty." But things are steadily, if slowly, improving, and we should be glad indeed if more workers would recognise that the sick and the infirm in workhouses, as well as in the more attractive hospital ward, have very strong claims on the services of those who are competent to give sorely needed skilled nursing.

### A SAD TRAGEDY.

A peculiarly sad incident was recorded last week at Eastbourne. An Irish gentleman's wife was undergoing "moral and spiritual" treatment, under Dr. Sherrard's care, at Avalon House, when she managed to push her nurse out of the room and lock the door, being afterwards found to have "fallen from the window." The poor lady was said to have been suffering from "depression of spirits" for some time past, and on one occasion she got on the roof of a house at Dorking, and threatened to throw herself off; she had also jumped through a window, and quite recently attempted to cut her throat. After the last occurrence, the doctor said at the inquest that he "instructed the nurses not to allow the deceased out of arm's reach." No evidence was however offered to prove that due precautions for the poor lady's safety were taken, nor was any explanation given as to the fact of a key being left in the lock of the door of the patient's room. The diagnosis, "she was suffering from morbid vanity and selfishness," is hardly sufficient to account for the repeated attempts at suicide; selfishness, at any rate, is not uncommon enough to rank as an abnormal characteristic!

### INDIAN HOSPITALS.

The beds in the new Albert Victor wing of the Mayo Hospital at Lahore, which was opened in February, have been incessantly occupied ever since by European and Eurasian patients. Natives have also been admitted in large numbers to their own portion of the hospital, which they evidently now regard with favour and confidence. They do not, however, contribute adequately to its support, and their subscriptions last year only amounted to Rs. 199, whilst the Europeans gave Rs. 759. It looks as if the wealthy native gentlemen of Lahore hardly appreciate the services rendered by the hospital to their poor countrymen.—At Rangoon a new brick building on the pavilion system has been planned as an extension of the Civil Hospital, which will secure accommodation for 84 paying and 300 native patients.

### A NURSE'S BIRTHDAY.

"What do the flags mean?" said an early riser one October morning as he looked at the gay bunting in Battle Harbour, Newfoundland. The fisherman addressed looked surprised at the question. "Why don't you know that its our nurse's birthday—God bless her!" Banished from civilisation for long weary months, living face to face with hardship, sickness, and death too sometimes, the toilers who supply us with one of the most useful forms of nourishment are keenly appreciative of the medical aid and the nursing skill which has reached them. There is more than a suggestion of poetry as well as the plain prose of gratitude in the feeling which prompted that "nurse's birthday" should not pass without the highest compliment which it is in the power of the sailor to pay to the man or woman whom he desireth to honour.

### SHORT ITEMS.

The West London Mission gave a grand conversazione the other evening at St. Martin's Town Hall, at which some nurses were present, this annual gathering being a notable event for London Methodists.—The Indian Government has granted a monthly allowance of 20 rupees to each lady of the Indian nursing staff during the time she is on field service, provided she keeps a horse or pony.—The Ophthalmic Committee in connection with the Central London School District carried, by nine votes to three, a proposal to increase the pay of two nurses who have done valuable service under the Board for a considerable period.—An entertainment was recently given to the nurses of the Glasgow Royal Infirmary by the Dennistoun Amateur Minstrels.—It has been decided to have a nurse for the sick poor of Ballycastle.—An association for supplying a trained nurse to the sick poor in their own homes has been formed at Henlow, Bedfordshire, and the committee hope to secure a Queen's nurse.—At Bramley the District Nursing Association has done excellent work, and has decided to engage a second nurse to work with Miss Roberts of the Q.V.J.I.

### On the Nursing of Diseases of the Nervous System.

### V.—CAUSES OF PARAPLEGIA.

The causes of paraplegia may be roughly divided into two groups, those which commence in the spinal cord, and those which begin outside the cord, and pressing upon it, destroy its functions. The most important in the first group is Myelitis, or inflammation of the spinal cord, of which there are several varieties. It may affect the whole thickness of the cord for a short distance, causing a complete break in the conducting paths, this is known as transverse myelitis, and usually occurs towards the lower end of the cord; or the inflammation is diffused through a considerable distance in the cord, this is the most serious and rapidly fatal form; or only a small area of the transverse section of the cord is affected, as in infantile paralysis. The first is the most common variety, and is due either to exposure to cold and damp after extreme exertion—for example, lying down on damp grass after a long walk—or it follows acute specific fevers, especially typhoid. There is generally a definite history of sensory symptoms before the paralysis is complete. The patient in the first-named example is wakened from sleep by sensations of tingling, or pins and needles, or actual pain referred to the part of the body in connexion with the particular portion of the spinal cord affected. These sensations are succeeded by numbness and after a period varying from a few hours to one or two days a complete paralysis ensues. When fully developed the patient will present all the symptoms of paraplegia, but in this case the loss of motion and sensation will be complete in the parts below the level of the damage, and there is usually just above the numb area a region of excessive sensitiveness. It is in this form of paraplegia that bedsores and bladder trouble are most likely to supervene, and may lead subsequently to a fatal termination. Death may also occur from inflammation of the cord spreading and paralysing the muscles of respiration. The patient may recover some power, although the limbs usually remain stiff, or he may live for years with completely paralysed and flaccid limbs. There is no special treatment for these cases, but bedsores and the bladder trouble must be carefully attended to by the nurse. Paraplegia arising in the cord from tumour is rare. Paraplegia arising from compression of the cord is most commonly found in Spinal Caries or Pott's Disease. The body or bodies of one or more vertebræ are diseased, and give way, and their spinous processes are displaced backwards, and form an angular deformity. The fewer the number of vertebræ involved the sharper is the angle formed by the projection; if a great number be diseased there will be a more rounded curve, in proportion to that number. In spite of the deformity, it is only very rarely that the pressure of the displaced bones gives rise to the symptoms; but usually the products of inflammation, form a kind of chronic abscess between the bone and dura mater, which projects into the spinal canal, causing compression of the cord.

Pott's disease is most common in children who are ill-fed and poor, and who live in towns. It is a scrofulous or tubercular disease, and other evidence of tubercular disease may probably be present, such as disease of the lungs, joints, &c. One of the earliest symptoms is pain, caused by the inflammatory swelling irritating the nerves as they come out of the spinal canal, and the pain is referred to the parts of the body where these nerves are distributed, and not commonly to the seat of disease itself. The middle or lower part of the back is very often affected, and the nerves coming off from the cord in this region pass round the body and end in the skin over the front of the abdomen, and hence the pain is felt here, and the child may be supposed to have stomach-ache. In all cases of persistent pain in the stomach the back is examined, and some tenderness over the spines of the vertebræ may be found on pressure, or some part may be more acutely sensitive to a sponge wrung out of moderately hot water, or coming down suddenly on the heels may cause pain in the back. Similarly, if the disease be higher up, the pain referred to the side of the chest may suggest pleurisy. Another effect of pressure is a certain amount of weakness, loss of power, and stiffness in the limbs, but suitable treatment may check the disease. In other cases the long-continued pressure may cause complete transverse myelitis. If complete rest be given to the spine the inflammatory products may disappear, and with that the cause of the compression, and there may be no alteration in the prominence of the back. The treatment is directed towards giving rest and support, so that a process of healing may go on in the diseased bones; but if part of the vertebra has been destroyed by disease it cannot be restored, and the deformity will remain. Rest by the application of a Sayre's jacket or extension are the means generally used. The Sayre's jacket will come well down to the hip-bones to give support, and if the disease is in the neck region the "jury mast" will be used. Cod liver oil and tonics are usually ordered, and general hygienic measures adopted. It is most important in these cases to prevent the ankle joints becoming stiff. Owing to the loss of power and the weight of the bed-clothes the toes point, and the foot tends to become fixed in this position; then the patient may recover power and yet the limbs be useless for walking, as the feet cannot be put flat on the ground. To avoid this a cradle is used, the ankle joints are bent and extended, and the feet turned in and out, the movements being gently performed for several minutes at least twice a day. The importance of this cannot be over-estimated. Recently in some cases of paralysis from Pott's disease an operation has been performed, and the cord freed from the pressure which is causing the paraplegia (Laminectomy); but the general applicability of this operation has yet to be determined.

*Malignant Disease* is another cause of compression paraplegia, but is rare. It may be of the nature of cancer or sarcoma, and in some cases an operation may successfully remove the cause of the compression, or there may be one or more similar growths in other parts of the body. In all cases, unless the disease can be completely removed, it ends fatally.

### Council Meeting of the R.B.N.A.

The first council meeting of the session was held by the Royal British Nurses Association on Friday, October 27th. There was not a large attendance. Her Royal Highness Princess Christian, Mr. Brudenell Carter, Mr. Langton, and Dr. Schofield were amongst those present. Mr. Pickering Pick was in the chair. The report of work done during the last term was read by the Secretary, Miss Daisy Robins. The Hon. Secretary, Dr. Bezley Thorne, then gave a report of the numbers who had joined, withdrawn, or died, viz., forty new members enrolled, thirty-two old members withdrawn, and six had died. A proposal was made to inaugurate a course of lectures for the benefit of members, other persons to be admitted on payment of a small fee. This motion was put and carried unanimously.

Sir Crichton Brown and Mr. Pickering Pick were re-elected as vice-chairmen, and the treasurer and hon. secretaries were also re-elected. The Hon. Secretary then read out a list of officers for re-election, and they were proposed by Princess Christian, and seconded by Sir Dyce Duckworth, and elected unanimously. The question of nurse members advertising themselves by recommending pills and quack remedies was mentioned in the course of the proceedings, and will, no doubt, be strongly discouraged by the Council. The meeting, which lasted nearly an hour, was dissolved, after awarding a vote of thanks to Mr. Pick.

### Nursing in the United States.

### PROPER ORGANISATION OF TRAINING SCHOOLS.

(By Miss Darche, Lady Superintendent New York Training School for Nurses, Blackwall Island.)

In this present age of charities and philanthropy, when the care of the sick, the insane, and the destitute, occupies so prominently the thought and attention of a benevolently disposed public, the building of hospitals and the management of hospitals has become one of the leading questions of the day. The improved methods of caring for the sick, as introduced by the training-school system of nursing, is generally acknowledged, and I think rightly acknowledged, as one of the prime factors in bringing about the wide-spread and intelligent interest now so generally felt in hospital work.

With the higher intelligence and skill introduced by this method of nursing, came the knowledge of higher possibilities of hospital development. Doctors were not slow to grasp the idea that a system and science in the treatment of the sick could now be introduced which before, owing to the low order of intelligence and the poor education of the old-time nurse, was impossible; that accurate reports, exact obedience, cleanliness and order came with the new nurse, and that inquiry and research into new fields could now be ventured upon, based on a knowledge gained from treatment faithfully carried out, and as faithfully recorded.

This improvement in the nursing when brought about, first received the notice and then the encouragement of the attending physicians and surgeons under whose observations the trial was first made. The change also soon made its impression upon the hospital authorities when it was demonstrated that where noise and unseemly altercation had prevailed in the management of "unruly patients," peace and quiet now reigned, and the so-called unruly patient seemed to disappear and become a thing of the past simultaneously with the disappearance of the old-time nursing and the old-time nurse.

The news of this improvement in the condition of hospital wards and the care of the hospital patients as a result of the introduction of the training-school system, in a very short time became generally known to the outside world. The friends of the patients told of it, visiting ladies and others interested in hospital work reported it, the visiting physicians and surgeons constantly commented on the improved results as shown by hospital statistics, and made favourable mention of the new reforms brought about by this improved system of nursing.

In this way and from its very beginning we see how the training-school system found favour on all sides, and it is not difficult to trace the vital influence and bearing which it has exerted in helping to make so popular as it is to day, the question of hospital management and hospital care of the sick.

In considering the subject of training-school management or organisation in this country, it will be well to try and understand the demand or great needs which first called this system into existence here; second, to understand some of the difficulties which had to be surmounted and overcome at the very outset of the undertaking; and, third, to trace how in overcoming these difficulties and obstacles the fundamental principles which underlie all good training-school government were gradually and experimentally worked out and finally established.

The first fundamental principle and starting point of all training-school organisation is the fact that hospitals exist, that they contain patients, and that the patients require nursing.

When the ladies who inaugurated the first training school in America formed into a committee for the purpose of starting a school for nurses, it was not because of the need of a school as a school for nurses, nor was it for the purpose of creating a new field of labour for women, a profession; but simply and solely because of the great need of one of the great charity hospitals for better nursing. They had visited the hospital, they had seen the misery and disorder, and they decided that any hope of reform must depend upon introducing into the wards as nurses women of an utterly different stamp from the nurses already there. But how to do this was a question which at first sight did not seem an easy one to solve.

They readily appreciated the fact that no self-respecting woman would go into the hospital and nurse under the then existing condition of affairs, to sleep where the old-time nurse slept, to eat what the old-time nurse enjoyed, to work under the then hospital officials, and to be treated with as little consideration and respect as the nurse of that time expected to be treated and deserved to be treated.

The idea of a separate administration in the management of the nurses, from that of the hospital administration, presented itself as the only way out of this difficulty. The hospital authorities were visited, the matter laid before them, and a contract entered into which placed the responsibility and management of the nursing of several wards in the hospital in the hands of what has proved to be the first Board of Managers of the first training school in America.

As this Board of Managers was composed of women from the first families in the country, the moral support and backing necessary to the undertaking was at one stroke accomplished. The idea of a home for the nurses, separate and away from the hospital surroundings, was at once thought of and provided. A few venturesome women of the right fibre, some of them actuated by a missionary spirit, consented to go into the work and engage as nurses; a superintendent was secured, and the enterprise started.

There were three distinct features of training-school organisation mapped out, viz.: (1) Hospital needs were met in providing hospital patients with conscientious and intelligent women as nurses. (2) The nursing had been placed under a separate management and head, with a distinct aim and purpose. (3) A home for the nurses had been provided. It will not be difficult to trace from this beginning how the training-school idea was gradually developed along the lines of the fundamental principles thus laid down.

The separate management of the nursing department, first brought about because there seemed no other way of getting the proper control of the situation, has proved to be the corner-stone of all training-school organisation. The contract entered into by the hospital authorities and the Board of School-Managers, at once indicated that the relationship between hospital management and nursing management could be established on a friendly, and at the same time on an independent, footing; and it was soon proved that the nursing under a distinct management and head could, and did, work harmoniously with the hospital management, and the mutual benefit and advantage of both.

Starting with this basis to work upon, the Board of Managers of the school found an ever-widening field of usefulness opening up before them. Under the wise direction of their representative-head, the superintendent, new methods of sick-nursing and of ward-management were introduced; the staff of nurses was increased to keep pace with the additional work entailed by the new methods; with the increasing popularity of the new movement, more wards were placed under the school management, and more nurses were added.

As new nurses were admitted, the older and now more experienced naturally assumed the position of head nurses and ward instructors. The intermediate nurses were called seniors, with duties accordingly, and a system of graded responsibility based on experience and merit began to take shape. In addition to the practical instruction in the wards which went hand in hand with the accomplishment of the daily nursing, the Superintendent started a system of class instruction, which was later supplemented by a course of lectures on the theoretical aspect of a nurse's work given by the visiting physicians and surgeons of the hospital. Thus the school idea in this scheme of nursing began to gain greater prominence as it was found that not only were the patients better cared for by reducing nursing to a system, but, also that in the system and through its operation, women were being daily trained as nurses; that the work itself, accomplished by proper methods and under proper supervision, had an educative value, this educative value becoming more apparent as the nurses advanced in their course of training and became more efficient in their work.

### Nursing in Newfoundland.

(By Our Own Correspondent.)

### MISSION TO DEEP SEA FISHERMEN.—LABRADOR.

We have been so busy that we have failed to carry out our good intentions to write full accounts of our work out here. We know the friendly interest and kind thoughts which have followed us, and therefore instead of wasting time in apologies we will let The Hospital readers know something of our doings since we reached Labrador.

First of all we must tell you that the arrival of The Hospital journal is a most welcome event, and we thoroughly appreciate being kept in touch with matters of which we should be otherwise ignorant. Pioneering work presents many difficulties, and of actual hospital experiences we have not a great deal to say as yet. We were well received at St. John's, and came on thence in the "Albert" to Battle Harbour, two doctors following in the "Princess May." The weather was foggy, but we had a service on board every evening, and the American organ was a great success, our ship having made the shortest passage on record, of which we were all very proud.

Our first sight of Battle Harbour will not easily be forgotten by us, for the morning was brilliantly fine, the sky a glorious blue with fleecy clouds, and the horizon marked by most delicate shades of purple. On the sea, which was a deeper blue than the sky, there floated 140 icebergs! We counted 117 on one side of our vessel.

The land appeared as a mass of rocks of a smoky hue, with just a dash of green here and there, altogether a very desolate looking shore.

We could just make out a flagstaff and a little church, the only one on the coast. The narrow entrance to the harbour is edged by little hills of 200 to 250 feet high on either side. At the foot of these cluster the houses built of wood.

The hospital, a good-sized four-roomed house, still required much to be done to it, as it was minus a staircase and chimneys, and lacked windows, partitions, &c. Our crew did a great deal for us, and meantime we visited the islands around, going from hut to hut. The doctor was generally addressed as "man," whilst "woman" was the title deemed sufficient for the nursing sister. We found and looked after two children with diphtheria.

Two of our party took up their residence in the roomy house of the agent, and visited the sick poor in their own homes whilst awaiting the completion of the hospital.

On our way further north in the "Albert" we called at many places, and in one house found a woman lying ill on a bed on the floor. She was placed near the fire, which was burning on the hearth, and down the wide chimney the wind blew in strong gusts. Two pots hung over the fire in which were cooking soup and peas, and two or three salmon were hanging up to dry. There was only one very small window, and a partitioned-off corner just gave space for a bed, which was, of course, in complete darkness.

(*To be continued.*)

### Nursing in Japan.

### TOKIO.

Japan is a country possessed of so many attractions that it is little wonder that nurses should from time to time discuss the idea of going out there, asking preliminary questions as to the prospects awaiting English women.

It is with great pleasure that we find ourselves able to give our readers reliable information from residents in the country who have kindly written as follows:—

A Nurse's Prospects in Tokio.

"In The Hospital of June 10th, 'Nurse F. S.' asks what prospect there is of getting employment in a hospital or in private families in Yokohama. I am writing in hope that she may see this before making arrangements to start. I have been living in Tokio for the past five years in charge of the medical work in connection with St. Hilda's Community Mission, and therefore I am speeking from personal knowledge. There is absolutely no hope of any work for a nurse in Japan. It is true there are many hospitals, and the number is increasing; but they are entirely and solely under the control of Japanese doctors and committees, and no foreign nurse is ever employed in them. When the Japanese first started hospitals they engaged nurses from Europe and America, but long before their time of contract was finished they were discharged. These nurses one and all tried for work in other hospitals and in other towns without success. They could not find employment of any kind whatever, and one or two had to be sent home by subscriptions raised by their own country people. At times there are severe cases of illness amongst foreigners, and then the patient's friends occasionally inquire if a nurse can be found; but this happens only once a year, or once in eighteen months. There is not the least likelihood of any nurse being able to make a living.

Only about four months ago a very accomplished gentlewoman holding first-class certificates and testimonials arrived in Yokohama and tried her utmost by means of introductions which she had to some of the doctors to obtain work, but entirely without success. When strangers are ill, they can now, by applying at any of the hospitals, obtain very fairly good Japanese nurses, and the foreigners as a rule prefer engaging the Japanese women, because they are much cheaper than either Americans or Europeans. The charge for a Japanese nurse is $2.50 to $3.50 a week, with everything found, and they eat the food of the country, which is a great convenience. They associate with the servants, and therefore the expense of their maintenance is very inconsiderable, whereas a foreign nurse must charge at the lowest $1.50 a-day, with special food, because when out of work she could not possibly live for less than $1 a day in the very cheapest lodgings. I have written plainly and fully, because it is a very serious thing for any woman to come out and experience the anxiety of money steadily decreasing, and no hope of making more. In fact, starvation stares any Englishwoman in the face who expects to make her own living in Japan, either by nursing or any other occupation."

A Nurse's Prospects in Yokohama.

"Under the heading 'Everybody's Opinion' in The Hospital of June 10th, an inquiry appeared respecting the prospects of English nurses in Japan. The writer spoke of intending to go out to Yokohama early next year; perhaps, therefore, the results of experience gained during a three months' visit to that country may be of interest to other readers as well as of special service to 'Nurse F. S.' The chief part of my time having been spent in Yokohama itself, I took the opportunity of visiting all the hospitals in the neighbourhood, and I interviewed many doctors, to whom I carried introductions. My wish to settle some workers there soon received discouragement. The hospitals proved to be quite impracticable, as natives are naturally better suited to attend on Japanese patients. They have far too great a dislike to 'foreigners' for them to offer Englishwomen any posts worth having. The low salary, which means wealth to a Japanese girl, seems so inadequate to trained nurses that it would be looked upon as merely nominal payment for the services required. In the naval hospitals no women are employed, nor are they likely to be demanded for those establishments. Private nursing might be lucrative if it were not too fitful for serious contemplation. There is, in fact, rarely any necessity for private nurses at the usual English or American rate of fees. Europeans appear to take very kindly to the Japanese nurses, who have received a fair training in some of the Tokio hospitals; they are particularly gentle, kindly, obedient, and willing to do all kinds of work, whether for the patient or for the household. They are contented with very small payments, and in this, of course, foreign nurses cannot attempt to compete with them. The expenses attendant on living in Yokohama are to strangers very considerable; the climate is most trying, and the intense heat in summer renders nay work somewhat of a hardship. A trained nurse going out to Japan to make her home with friends or relations, or one with private means, would probably get an occasional patient amongst the residents who prefer to be attended by their own countrywomen, but such work could never be definitely reckoned upon. Of course, that ideal employer, the wealthy American, might be taken ill when visiting Yokohama and prove a remunerative patient, but such a case is uncommonly rare. It appears, therefore, a grave blunder for any Englishwoman, however well trained and experienced, to seek fortune or even 'a modest competence' by means of a career as a hospital or private nurse in Japan."

### A Bird Show.

(By a Nurse Who Does Not Wear Feathers.)

It may interest nurses who take an interest in pets to hear of a very good show of canaries and British and foreign birds, held in the Royal Aquarium, Westminster. The exhibition, which lasted three days, was opened on October 27th. Entering the hall of the Aquarium by a side door we are at once surrounded by feathered songsters of every shade, from the palest primrose to the deeper copper-colour. Until brought face to face with a collection of canaries such as this, it is impossible to realise the great variety of form and colour which exists amongst these birds. For instance, we saw one class with hump-backs, much resembling our old friend Mr. Punch, contrasting strangely with the long slim forms of their neighbours. Again, the crested Norwich formed a particularly fine class, though to the inexperienced eye their little heads at once suggested miniature mops. They looked like birds that had been hurried over their toilets without time for due attention to the arrangement of their feathers. Their next door neighbours displayed notably smooth and glossy polls. There were some beautiful creatures in class 71, which consisted of a variety of red-coloured birds, and our admiration was also claimed by the fine specimens in class 63 of Norwich crested canaries. Amongst the small British birds we noticed kingfisher, red-polls, and a variety of tits, Dr. J. D. Bradburn exhibiting the red-poll that took second prize in last year's show. Altogether the eighth exhibition of the London and Provincial Ornithological Society proved well worth a visit, and should do much to soften the heart of Queen Fashion. How much more gracefully the plumes are worn by the rightful owners than by those who, considering that might is right, ruthlessly encourage the annual destruction of thousands of beautiful little creatures for the sake of their feathers.

### Women's Work.

### I.—Choice of a Career.

Every woman has her mission, although she may fail to accomplish, or even to realise what it is. There is work waiting for each one in some quarter of the globe; in fact, there are home and foreign missions for all of us. Naturally the first ought never to be neglected for the last, and yet there is no reason why the two should not march comfortably together. The old theory that girls must be content within the narrow walls of home, growing into middle-aged, often discontented women there, sacrificed to the masculine decree that it was their only proper sphere, is now, happily, obsolete. Those who succeed in their home missions generally do well in foreign, *i.e.*, missions beyond the family circle.

When there are several daughters in a household they are seldom all needed, and inevitably there arises a question of definite outside work for one, at least, of the number. A family which spares one member for the service of the public at large itself derives benefit from the circumstance. New interests from other work and other lives come into the circle. At each visit the stray daughter brings news of her special surroundings. Even when she has quitted the nest under protest, as it were, she invariably finds, as time goes on, that she is regarded with affectionate pride by her family.

But in leaving home, whether voluntarily or of necessity, a girl should strive for a definite career. She should not fancy that "getting something to do" is her only duty. She should aim at getting work both congenial and useful.

Once there seemed nothing save teaching or "being a companion" for the aspirant. To-day she has almost as wide a choice as her brothers. For instance, the pioneer medical women were once pelted with stones and with ridicule, whereas they are now a recognised power in the land.

The first trained nurses had experiences which the probationer of to-day would shun with scornful disgust.

Unless girls have ability and special taste for the profession of medicine or the art of nursing, they had better not attempt either. Neither should they join any mission until they have "counted the cost" of doing so.

To go forth as teachers, whether of religion or science, they must qualify themselves, and, therefore, in choosing a career, due preparation for it must not be lost sight of for a moment.

If medical missions prove attractive, then girls must bravely face the long course of training by which alone the goal can be reached. They must go through the whole curriculum, and not dream of adopting disastrous half-measures. If they have neither time nor money for doing one particular thing, then let them look round and find another less costly outlet for their talent and energy. Only whatever is attempted let it be completed.

Many failures would be avoided and much heart-sickness if women considered the suitability of certain occupations in their own case. There is so much work in the world waiting to be done, but it should not be picked up haphazard. We owe respect to the duties we take upon us as well as to ourselves. According to their fulfilment we either benefit or injure others. All work is worth doing well, and should therefore be undertaken with some preconceived knowledge of what it will require from us.

Even the cleverest fingers have to be taught how to turn the heel of a stocking, and workers, whether philanthropists or bread-winners must fail without instruction in their subject. Whilst in England it is comparatively easy for a woman to turn from one occupation to a new thing until she eventually finds out a congenial vocation. Yet for those taking up work in distant lands it is advisable that they should prepare themselves most thoroughly before going out. Whatever difficulties beset a career in our native land, we have always help within reach, whilst in colonies and foreign parts it is often quite unattainable.

All who can be spared from home, whether for a time or permanently, should certainly strive to find work fitted for their tastes and talents, because it will be far better done than if it were distasteful, and above all things it should be an occupation leading to some useful, and therefore satisfactory end.

### Where to Go.

The Central Conference of Women Workers will be held at the Albert Hall, Leeds, on November 7th, 8th, 9th, and 10th.

Lecture.—The next lecture at the Midwives' Institute and Trained Nurses' Club will be given on Friday evening, November 17th, at a quarter to eight, by Dr. A. D. Leith Napier. Subject: "A Glance at Some Obstetrical Topics."

The Obstetrical Society of London.—The next examination of candidates for the diploma of midwife will be held on Wednesday, January 10th, 1894. For particulars apply to the Secretary, L.O.S., 20, Hanover Square, W.

The next set of classes for preparation for the L.O.S. Examination will commence on Thursday, November 2nd, at five o'clock. This class is held at the Midwives' Institute. For particulars apply to Mrs. Nichol, 12, Buckingham Street, Strand.

Popular Lectures at Toynbee Hall.—On Saturday, November 4th, at eight p.m., Mr. W. M. Conway, on "Climbing in the Himalayas"; and on November 11th Colonel Sir Colin Scott Moncrieff will give a lecture at the same hour.

The Imperial Institute.—The Prince of Wales has sanctioned the following arrangements made by the Executive Staff for the Winter Session of the Imperial Institute, which will be opened by the Prince of Wales on November 20th, when Professor Leckie delivers a lecture. On November 22nd and alternate Wednesdays there are to be smoking concerts in the Grand Hall, which has been fitted up for the purpose. Male artistes will be engaged, and refreshments will be provided at the tables. On the intermediate Wednesdays there are to be ladies' concerts, the hall being then arranged without tables, and no smoking will be permitted. The Prince of Wales will, it is expected, inaugurate both of these series of entertainments. On other days of the week lectures will be delivered on technical and commercial subjects, and on Monday evenings special illustrated lectures will be given. It has been also determined to at once establish a distinct orchestral and vocal society, which will be conducted by musicians of eminence. It is arranged that at the smoking concerts only first-class vocalists will appear. Ladies will be admitted to these entertainments, but there will be only male performers. The Great Hall is capable of accommodating over two thousand persons, and it is anticipated that these meetings will become extremely popular during the winter months.

### Appointments.

Bridport Cottage Hospital.—Miss Catherine Marshall has been appointed Matron of this hospital. She was trained at Oldham Infirmary, worked as Charge Nurse at Southport and Carlisle Infirmaries, and as Senior Nurse to the Kettering District Association. Miss Marshall was formerly Matron of the Boys' Home, Eardisley, and also Parish Nurse. We wish her every success in the new work which her varied experience has specially fitted her for.

### Notes and Queries.

### Queries.

(213) *Europeans.*—Will anyone give me information about the Association for Nursing Europeans in India?—*R. K.*

(214) *Dispensing.*—Where can I learn dispensing.—*Ex-Nurse.*

(215) *Co-operation.*—What is the address of the Nurses' Co-operation in London?—*S.H.*

(216) *Eyes.*—Can anyone recommend a useful book on ophthalmic nursing, to a trained nurse?—*F.G.R.*

(217) *Monthly Nursing.*—Wanted the name of book on midwifery.—*L.C.H.*

### Answers.

(213) *Europeans* (*R. K.*).—See The Hospital of April 29th, 1893, page xlvii.

(214) *Dispensing* (*Ex-Nurse*).—This subject is fully explained in "Burdett's Annual," which can be obtained of the Scientific Press, 428, Strand.

(215) *Co-operation* (*S.H.*).—Address Lady Superintendent, 8, New Cavendish Street.

(216) *Eyes* (*F.G.R.*).—A book on ophthalmic nursing by Sydney Stephenson has been recently issued by the Scientific Press, price 3s. 6d.

(217) *Monthly Nursing* (*L.C.H.*).—A new book will be brought out shortly by the Scientific Press.

### For Reading to the Sick.

### MISUNDERSTANDING.

We are all quite agreed that it is very unpleasant to be misunderstood and declare those persons to be perverse or stupid who do not take our words and actions in the way they are meant. But let us turn the situation round and look at the other side. Are we as clear sighted as we might be with regard to the dealings of the Almighty with us? Are we not frequently perverse, or stupid, or even worse, deliberately setting ourselves against the rulings of providence; often "nursing our wrath to keep it warm," when we should be trying to reconcile ourselves to our lot? Our lot, perhaps, seems to be one of suffering, and we cannot bear that it should be so, while others are happy and enjoying themselves. Now our Heavenly Father does not love to chide, but He acts towards us as a judicious earthly parent would do by his family. A good father would give his children naught to harm them though they cried for it, nor let them play on the edge of a precipice without warning them of the danger. The little ones in their ignorance complain of the restrictions set upon their liberty and try to escape from them, so that the father is compelled to tie them up within bounds, or remove them from the scene of temptation.

It is just for the same reasons that God sends us trials and sickness. We are thoughtless, and run our souls into unnecessary dangers; ungrateful, and slight the blessings He heaps upon us. We are not contented with the simple pleasures of health and food and raiment, nor with the wise Agar pray, "Give me neither poverty nor riches, feed me with food convenient for me"—but we make haste to be rich, or throw ourselves violently into our pastimes, and our health breaks down with the strain. Then we complain of our afflictions, and think our Heavenly Father is dealing hardly with us. Let us have no misunderstandings with the Almighty. Our Father always acts by natural means, and all the grumbling and discontent in the world will not prevent the consequences of a wrong or foolish action. If we are wisehearted we shall ponder in our hearts the causes of our sufferings, and we shall see that they have come upon us for our good; they have pulled us up short in a course of folly, or they have checked us in an inordinate desire to make money any way; they are teaching us to "Seek first the kingdom of God and His righteousness." All other things will be sent for our benefit and happiness if we take patiently the ills we know of. When our dear Lord sees that we are striving to follow His example, to drink the bitter cup from our Father's hand without a murmur, He will send an angel to strengthen us. His own hand will still our throbbing brows and moisten our parched lips when, without misunderstanding God's providence we can say "Thy will be done."

### Everybody's Opinion.

[Correspondence on all subjects is invited, but we cannot in any way be responsible for the opinions expressed by our correspondents. No communications can be entertained if the name and address of the correspondent is not given, or unless one side of the paper only be written on.]

### NURSING AT JOHANNESBURG.

"Nurse P." writes: Enquirers about nursing in other countries seem answered so fully in The Hospital that I venture to ask now if anyone can give me information as to a nurse's chances in Johannesburg? I should be very grateful to those readers who would tell me if thoroughly trained monthly nurses are required there.

### Wants and Workers.

Nurse E. has several trusses almost new which she will be pleased to send, carriage paid, to any district nurse for the use of poor patients.

### A Groat's Worth of Wit.

### ON SHORT STORIES.

It requires something of an effort for busy people to undertake a novel of the length of "Lorna Doone" or "Robert Elsmere," or the much-debated "Heavenly Twins." It takes some little time to get into the story at all, and when there is only an occasional half-hour to give to it, the design of the whole gets lost and the outlines of the characters blurred. It is perhaps for this reason and because the tendency of the age is to make everyone either busy or persuaded that they are busy (a much more fatal thing), that short stories are increasingly in demand, and have become the stock in trade of many notable writers. Like certain bonbons, which are labelled "to be put in the mouth whole," the short story must be read at a sitting at the risk of losing something of its particular flavour. If it is good it will bear reading twice, once for the story, and again for due observance of style and finish. The three volume novelist, in introducing weedy digressions, which serve no purpose, blinds many readers to the fact that in an artistic story no touch is superfluous.

The mention of short stories brings to everyone's mind the name of Rudyard Kipling. It would be difficult to find any English writer who has reached greater perfection in this art, and the explanation lies perhaps in the fact that he has thrown into it his full strength, instead of reserving it for a Christmas number. * "Many Inventions" is a wonderful piece of workmanship of endless variety, and incredible as it may sound—not one out of the eight or ten stories which make up the volume turns on the making of love. Mr. Kipling is almost as shy of women as R. L. Stevenson. But there can be no question that in courageously excluding the great luminary of fiction, as of life, he gains more than he loses. Just as certain processes of art demand the exclusion of sunlight, the full play of what may be called the minor passions demands the suppression of the overpowering force before which the common interests of life dwindle and pale. These "inventions," with their vivid mark of personal observation, might be more fitly called experiences. "In the Bush" gives, for instance, the strange passion of solitude in the presence of the myriad life and unfathomed mystery of the jungle, and might have been written on the spot, so full is it of the peculiar essence of a half-savage life. Then there is the lust for fighting and conquest exemplified in more than one story; the passions of honour, friendship, revenge; the humours of the elephant world (Mr. Kipling may be said to have discovered the elephant), and the marvellous dissection of a sick brain with which the volume opens.

In †"The Private Life" we are in an entirely different world at once. The languid ladies and gentlemen who make up the greater part of the *dramatis personæ* are not deeply interested in anything, and cannot be conceived likely to do or even to feel very acutely. But they are a graceful company, and talk uncommonly well. Their lives seem a trifle aimless, but they are so good as not to mind that particularly, and if their love affairs show a strange tendency to go wrong, all for the want, as it seems, of a little energy, they at least accept the situation with a good grace and marry the next comer without too much demur. The same apathetic atmosphere extends to "Owen Wingrave," which is an old-fashioned ghost story of the usual description, redeemed, however, by the subtlety and delicate touches of character which are never wanting in Mr. Henry James's work.

In the Pseudonym Library the short story finds a congenial *milieu*. But its latest addition, "Cavalleria Rusticana,"‡ will not add much to its reputation. Slight enough in the original, their own dialect must have lent the tales a certain charm as pictures of peasant life, but in the translation this charm has evaporated completely. It is probably not the translator's fault. Folk stories are as untranslatable in their way as Homer or Dante, and the retention of a few words of the dialect to lend them local colour will not redeem the baldness of the talk or replace the vanished essence of the old tradition.

No books about the Jews, not Lord Beaconsfield's or George Eliot's, have till lately succeeded in painting their daily lives and aspirations as they are. "Daniel Deronda" painted them as the authoress wished them to be. But Mr. Zangwill, in the "Children of the Ghetto," has introduced us into the very heart of the Jewish world, and aroused sympathy not for the impossible hero of the familiar Beaconsfieldian type, but for the striving, suffering, fiercely spurned alien and outcast, whose very existence in our midst stirs the wrath of the political economist, and whose character and habits are too often highly unedifying. The "Children of the Ghetto" is open to the objection that it is more a series of scenes than a connected whole. Broken into fragments, it would have more real unity than in a three volume novel. This suspicion is confirmed by the appearance of a small volume of "Ghetto Tragedies" § by the same author, in which the concentration demanded by the limit of the short story tells most favourably on the style. Satan Mekatrig is an exceedingly weird and powerful tale, and the insight it gives into the crystallised but still vital forms of the old religion as observed to-day in our midst, is even more amazing than its "flesh-creeping" qualities. The little sketch called "Incurable" is less ambitious, but it touches a deeper note of feeling. The scene is laid in an East-end refuge for Jewish incurables, and the humours of this little world where hope can never enter, but where love and sacrifice hover and make all beautiful, are depicted with terse vigour and a power of sympathy no outward degradation of form can repel.

* "Many Inventions." By Rudyard Kipling. (Macmillan & Co.)

† "The Private Life," &c. By Henry James. (James R. Osgood, McIlvaine, and Co.).

‡ "Cavalleria Rusticana." Pseudonym Library. (T. Fisher Unwin.)

§ "Ghetto Tragedies." By I. Zangwill. (Maclure and Co.)

### Novelties for Nurses.

### TOWNSEND AND COMPANY'S METAL WORKS.

Christmas is drawing near, and with it come the joys and the difficulties of Christmas presents. One and all desire that their gifts should be acceptable and appropriate to the receiver. We have just received a little catalogue of all sorts and descriptions of metal work, from the lowly match-box to the more pretentious lamp. The Townsend Company's wares we know to be good in quality, and some of the goods they offer strike us as being very suitable and useful presents for nurses. When a presentation is about to be made there is usually the difficulty of deciding on something that is both useful and of handsome appearance. We think we may help to solve the difficulty for some by giving an illustration of the "Whangee" standard kettle which lends itself to nurses and matrons' requirements, as it looks well, obviates the difficulty of a fire, and does not take up space on the tea table. It is made in the best polished brass at a cost of £2 5s., and is provided with a regulating safety spirit lamp.

### The Book World for Women and Nurses.

[We invite Correspondence, Criticism, Enquiries, and Notes on Books likely to interest Women and Nurses. Address, Editor, The Hospital (Nurses' Book World), 428, Strand, W.C.]

Woman's Mission. Papers on the Philanthropic Work of Women. Edited by the Baroness Burdett-Coutts. (Sampson Low, Marston, and Co.)

This cheerful large volume of beautifully clear type is a perfect encyclopædia of women's good works, and also most entrancing reading. The difficulty of compressing such an enormous amount of information on such a variety of charities into a volume so easily held that one can continue to read it for hours with unflagging interest, must have seemed to the Editors almost unconquerable. Their method is as unusual as it is successful. The Princess Christian requested Lady Burdett-Coutts to prepare an account of women's manifold charities which might be forwarded to the Women's Section of the Chicago Exhibition. A committee was at once formed to obtain information from the "heads of all religious communions, all the principal philanthropic, social, and charitable institutions, and also from those known to be working either in smaller bodies or even single-handed for kindred objects. It was requested that the information should be given not by printed reports, but written papers personally signed. These papers are bound into five large volumes, which are now at Chicago. The great difficulty that arose was as to how this mass of information could be most conveniently placed before the public of England, and the editor conceived the happy idea of of submitting the various batches of reports to accomplished women writers, who "boiled down" each subject and dished it up again in a digestible and palatable form. Thus "Hesba Stretton," the well-known novelist, tells us of "Women's Work for Children," Mrs. Molesworth of "Food, Fun, and Fresh Air for the Little Ones," Lady Compton of "Women's Work in Ragged Schools," Mrs. G. A. Sala of "Working Guilds." There are also chapters on "Clubs for Boys and Young Men," "Working Girls' Clubs," "Emigration" "Work among Navvies," "Responsibilities of Mothers," "Rescue Work among Women," "Work among Soldiers and among Sailors," "Sick Nursing," "Homes for the Dying," "Women as Guardians of the Poor," and many more too numerous to recite. In each chapter the history of the movement in question and its originators are given. It should encourage us all to do even the humblest amount of good that comes within our reach, when we read how small and how extremely simple were the beginnings of institutions which now interest the whole of the philanthropic world, and whose organisations are so complicated that they require paid officials to superintend them. Such, for instance, as the Ragged Schools Union and the Bridge of Hope Mission.

Lays of the Scottish Highlands, and Other Poems. By Ryder N. Breeze. (Ward and Downey.)

The versification of the longer pieces is not such as to justify the author in aspiring to the difficult region of narrative poetry. Some of the Prison Rhymes are simple and prettily expressed, and have an interest of their own, as containing presumably a record of personal experience.

**THE BOOK WORLD FOR WOMEN AND NURSES**—*continued.*

REVIEWS OF THE MONTH.

The Humanitarian, devoted to the laudable enterprise of improving the human race, does not omit the more immediate claim of improving itself. It has discarded the old disfiguring cover, and has enlisted excellent writers in the cause of temperance and education. The endless talk about woman's position is a little wearing. In view of certain writers who persist in regarding her as a newly-discovered animal, it is difficult to realise that she has been with us (a few days only excepted) since the creation of the world.

The Religious Review of Reviews tends necessarily to be rather heavy reading, but it no doubt serves a useful purpose in bringing notes of important contributions to the religious discussions of the day. It might, with advantage, be a little more comprehensive, and include some consideration of the Nonconformist world.

In the Revue des Deux Mondes, M. Mélinand discusses the knotty point, "Why do we blush?" It is amusing to find that the scientific opinions unite in considering this habit of blushing an entirely useless and even dangerous gift, so much so that it is quite inexplicable why the blushers, being evidently the unfittest, should have survived in the struggle for existence. M. Mélinand, analysing all the causes which produce blushes, concludes that they result from the fear of betraying some hidden sentiment; but this hardly explains why women more than men are given this way. Does M. Mélinaud believe that theirs is the larger share of hidden sentiments to conceal?

La Nouvelle Revue is bringing out a very striking series of articles by M. Delacroix on "Witchcraft Trials in the Seventeenth Centuries." The serious way in which "psychical research" was dealt with in those days is illustrated by a decree of the Parliament of Bordeaux deciding that trouble brought into a house by the occupation of ghosts is a sufficient motive for the cancelling of the lease. But deducting all that was the result of panic or malice, there remains an undeniable substratum of fact in the accusations against the unhappy creatures who practised the black art, and it can hardly be doubted that, by a species of what is now known as hypnotism, aided by the unquestioning belief in their powers shown by their neighbours, the witches were able to do quite enough mischief to constitute them a serious danger to society.

The November number of The English Illustrated Magazine is showing a steady improvement under its present change of management. The articles throughout the present issue are deserving of attention. Perhaps the chief amongst which is a short tale by the author of "Dodo," which evinces no falling off in the literary value of Mr. E. F. Benson's work. Mr. Clement Scott contributes a study of Japanese girls, which subject the writer has treated from a more dispassionate standpoint than has characterised other descriptions of young ladies of the Mikado's land. Mr. Scott shows us a Japanese girl as he has seen her "pudding-faced, greasy-haired, bandy-legged"—not the idealised creation of Pierre Loti's brain. "Reminiscences of Balliol College," by Andrew Lang, illustrated by Holland Tringham, and including a portrait of "The Master," has a special claim on our interest at this time.

## Figures and Tables

**Figure f1:**